# Identification of the genetic and clinical characteristics of neuroblastomas using genome-wide analysis

**DOI:** 10.18632/oncotarget.22495

**Published:** 2017-11-18

**Authors:** Kumiko Uryu, Riki Nishimura, Keisuke Kataoka, Yusuke Sato, Atsuko Nakazawa, Hiromichi Suzuki, Kenichi Yoshida, Masafumi Seki, Mitsuteru Hiwatari, Tomoya Isobe, Yuichi Shiraishi, Kenichi Chiba, Hiroko Tanaka, Satoru Miyano, Katsuyoshi Koh, Ryoji Hanada, Akira Oka, Yasuhide Hayashi, Miki Ohira, Takehiko Kamijo, Hiroki Nagase, Tetsuya Takimoto, Tatsuro Tajiri, Akira Nakagawara, Seishi Ogawa, Junko Takita

**Affiliations:** ^1^ Department of Pediatrics, Graduate School of Medicine, The University of Tokyo, Tokyo, Japan; ^2^ Department of Pathology and Tumor Biology, Graduate School of Medicine, University of Kyoto, Kyoto, Japan; ^3^ Department of Pathology, National Center for Child Health and Development, Tokyo, Japan; ^4^ Cell Therapy and Transplantation Medicine, The University of Tokyo, Japan; ^5^ Laboratory of DNA Information Analysis, Human Genome Centre, Institute of Medical Science, The University of Tokyo, Tokyo, Japan; ^6^ Saitama Children's Medical Center, Saitama, Japan; ^7^ Gunma Red Cross Blood Center, Japanese Red Cross Society, Gunma, Japan; ^8^ Research Institute for Clinical Oncology, Saitama Cancer Center, Saitama, Japan; ^9^ Laboratory of Cancer Genetics, Chiba Cancer Research Institute, Chiba, Japan; ^10^ National Center for Child Health and Development, Tokyo, Japan; ^11^ Japan Neuroblastoma Study Group; ^12^ Saga Medical Center Koseikan, Saga, Japan

**Keywords:** copy number variants, target amplicon deep sequencing, ALK, ALK immunohistochemistry staining, Japan neuroblastoma study group (JNBSG)

## Abstract

To provide better insight into the genetic signatures of neuroblastomas, we analyzed 500 neuroblastomas (included specimens from JNBSG) using targeted-deep sequencing for 10 neuroblastoma-related genes and SNP arrays analysis. ALK expression was evaluated using immunohistochemical analysis in 259 samples. Based on genetic alterations, the following 6 subgroups were identified: groups A (*ALK* abnormalities), B (other gene mutations), C (*MYCN* amplification), D (11q loss of heterozygosity [LOH]), E (at least 1 copy number variants), and F (no genetic changes). Groups A to D showed advanced disease and poor prognosis, whereas groups E and F showed excellent prognosis. Intriguingly, in group A, *MYCN* amplification was not a significant prognostic marker, while high ALK expression was a relevant indicator for prognosis (*P* = 0.033). Notably, the co-existence of *MYCN* amplification and 1p LOH, and the co-deletion of 3p and 11q were significant predictors of relapse (*P* = 0.043 and *P* = 0.040). Additionally, 6q/8p LOH and 17q gain were promising indicators of survival in patients older than 5 years, and 1p, 4p, and 11q LOH potentially contributed to outcome prediction in the intermediate-risk group. Our genetic overview clarifies the clinical impact of genetic signatures and aids in the better understanding of genetic basis of neuroblastoma.

## INTRODUCTION

Neuroblastoma is the most common pediatric extracranial solid tumor and accounts for approximately 15% of all pediatric cancer-related deaths [1]. The clinical course of patients with neuroblastomas is highly variable, ranging from spontaneous regression to widespread metastatic disease, mirroring the biological and genetic heterogeneity of this disease [1]. Despite intensive multimodal therapy, the prognosis of high-risk neuroblastoma still remains poor with a 5 year survival rate of approximately 40% [1, 2], and this underscores the importance of developing novel therapeutic modalities on the basis of the understanding of neuroblastoma pathogenesis.

In recent years, several genetic changes, such as *MYCN* amplification [3], loss of heterozygosity (LOH) of chromosomes 1p and 11q [4, 5], and 17q gain, have been identified in neuroblastoma specimens, which have been shown to correlate with its aggressive clinical features [6]. However, these genetic changes explain only half of the cases of high-risk neuroblastoma, and an overview of the genetic heterogeneity in all stages of neuroblastoma has not been fully presented. On the other hand, the discovery of *ALK* mutations/amplifications in approximately 10% of neuroblastoma cases represents a major impact on neuroblastoma research, as it reveals a novel molecular mechanism involved in the pathogenesis of neuroblastoma and provides a basis for the development of therapeutic strategies [7–10]. In addition, several groups have reported a strong significant trend between high ALK expression levels and poor outcome in patients with neuroblastomas [11, 12]. However, as the mutation/amplification rate in neuroblastoma cases is relatively low, our knowledge regarding the genetic profiles of neuroblastomas with *ALK* abnormalities is still limited. More recently, genome-wide searches for genetic alterations using whole-exome/genome sequencing have revealed that neuroblastomas, similarly to other pediatric malignancies, have very few additional gene targets, including *ATRX*, *TERT*, and *RAS* pathway genes [13–17]. However, no comprehensive study has yet explored the profiles of genetic aberrations and their potential contributions to clinical phenotypes in a large series of neuroblastoma cases. Therefore, to provide further insight into the genetic signatures of neuroblastomas and their contributions to clinicopathological features, we conducted a genome-wide study in a cohort of 500 neuroblastoma cases using targeted deep sequencing and single nucleotide polymorphism (SNP) array analysis combined with immunohistochemical (IHC) analysis of the ALK protein.

## RESULTS

### Division into genetic subgroups based on genetic signatures, ALK aberrations, MYCN status, other gene mutations, and copy number variations (CNVs)

**Figure 1:**
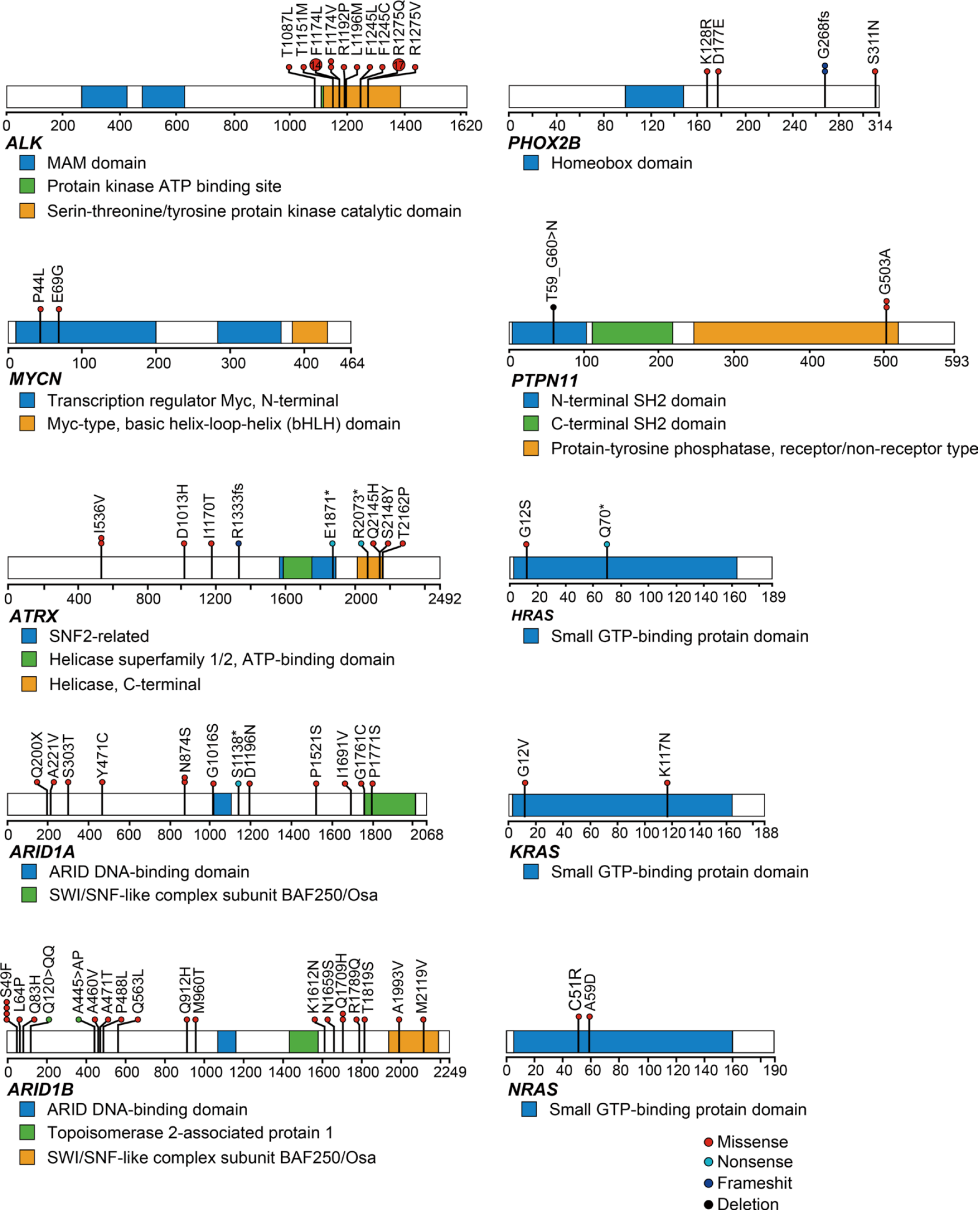
Mutational patterns of representative genes detected by targeted amplicon sequencing (*N* = 500) Mutation distributions for *ALK*, *MYCN*, *ATRX*, *ARID1A*, *ARID1B*, *PHOX2B*, *PTPTN11*, *HRAS*, *KRAS*, and *NRAS* in 500 neuroblastoma cases. Types of mutation were distinguished by the indicated colors.

**Figure 2:**
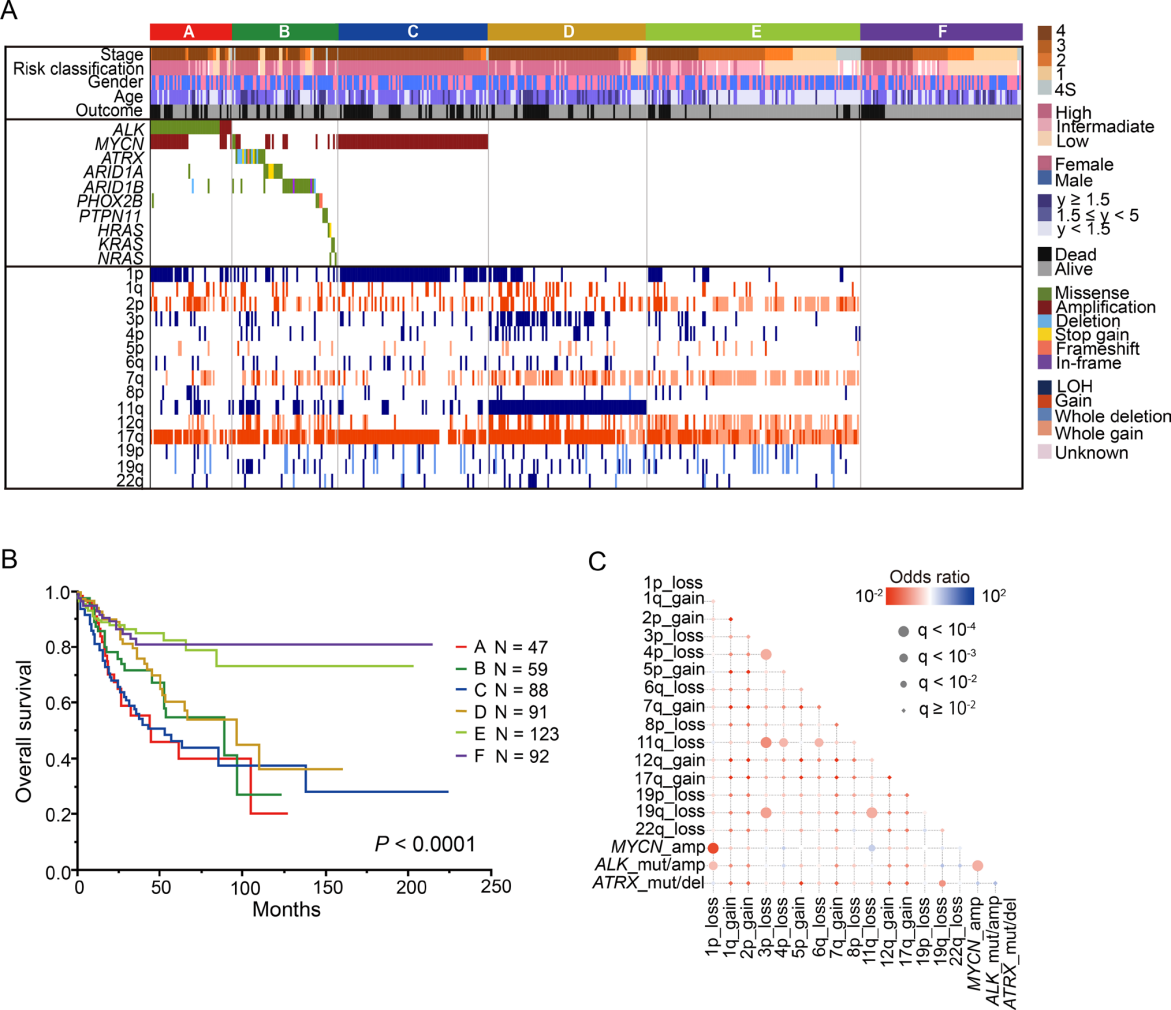
Genetic characteristics and overall survival of 500 neuroblastomas (**A**) Landscape of genetic lesions in the 500 neuroblastomas. Molecular classification, histological type, INSS classification, and risk classification together with the numbers of affected cases and the types of mutations and CNVs are shown by color as indicated. Based on the genetic lesions, neuroblastoma patients were divided into the following 6 genetic subgroups: group A, *ALK* mutation/amplification; group (**B**) other gene mutations without *ALK* mutation/amplification; group C, *MYCN* amplification and 1p LOH; group D, 11q LOH; group E, copy number variations without gene mutations, *MYCN* amplification, and 11q LOH; and group F, silent group. B. Kaplan–Meier overall survival curves for the 500 neuroblastoma patients according to the 6 genetic groups. (**C**) Significant positive and negative correlations among major driver alterations are indicated with their odds ratios. Red, gene mutation/copy number change pairs that are co-altered more than that expected by chance; blue, mutually exclusive gene mutation/copy number change.

We identified 102 mutations involving the 10 neuroblastoma-related genes in 98 of the 500 patients (19.6%) (Figure 1, Supplementary Table 1). Mutations were most frequently observed in *ALK* (9.4% of cases), and the mutations were concentrated around the hot spots in the kinase domain (Figure 1). The second most frequently mutated gene was *ARID1B* (4.4% of cases) (Figure 1, Supplementary Table 1). Additionally, *ARID1A* and *ATRX* mutations were recurrently detected in our cohort at frequencies of 2.4% and 3.4%, respectively (Figure 1, Supplementary Table 1). Although loss of function mutations or deletions of *ATRX* have been implicated in the pathogenesis of neuroblastoma, we found only 3 putative loss of function mutations in this study. Mutations in *PHOX2B* and *RAS* pathway genes were previously reported in 2.3% and 3.7% of neuroblastoma cases [18, 19], but such mutations were less frequent in our screen here (Figure 1, Supplementary Table 1). Although the mutation rate of the targeted genes was relatively low, considerable variations in copy number changes were observed in most neuroblastoma individuals (frequency, 81.6%). Recapitulating previous reports [6, 20–22], neuroblastoma genomes were characterized by common 17q gains with or without 1p, 3p, 4p, 11q, 19p, and 19q LOH, and 1q, 2p, 7q, and 12q gains with varying combinations (Figure 2A). As shown in Figure 2A, the CNVs found in more than 5% of the samples were 1p LOH (30.2%), 1q gain (13.2%), 2p gain (35.8%), 3p LOH (15.8%), 4p LOH (8.8%), 5p gain (7.6%), 6q gain (7.0%), 7q gain (36.2%), 8p LOH (5.8%), 11q LOH (28.2%), 12q gain (29.4%), 17q gain (73.4%), 19p LOH (6.6%), 19q LOH (7.4%), and 22q LOH (6.2%). Aberrant high expression of *TERT* due to intragenic rearrangements were frequently found in aggressive phenotype of neuroblastoma [15, 16]. These changes can be detected by fluorescence *in situ* hybridization and expression analysis, but unfortunately, since RNA and/or frozen cell samples were not available, we could not evaluate *TERT* abnormalities in this study.

The most prominent genetic signature of the entire cohort was the apparent presence of the following 6 genetic subgroups defined by common genetic changes (Figure 2A): (1) the group with *ALK* mutation/amplification (group A; *n* = 47); (2) the group with other gene mutations without *ALK* mutation (group B; *n* = 59); (3) the group with *MYCN* amplification without other gene mutations (group C; *n* = 88); (4) the group with 11q LOH without *MYCN* amplification and other gene mutations (group D; *n* = 91); (5) the group with CNVs without gene mutations, *MYCN* amplification, and 11q LOH (group E; *n* = 123); and (6) the group showing no genetic changes (group F; *n* = 92). As group F showed almost no genetic changes, we checked the histological criteria of 50 of the 92 tumors in this group, for which paraffin sections were available. All these tumors revealed a high number of neuroblastoma or ganglioneuroblastoma cells, indicating that purity was high in at least these 50 tumors, although the possibility of very low tumor content could not be excluded for the remaining samples. In this group, 14 cases died and 2 tumor samples from dead cases had poor quality due to low volume of tumor. All 14 tumors were taken before starting chemotherapy, but only one case could be re-checked histologically in the current study. Thus, tumor contents and quality of majority dead cases in group F were unclear.

Importantly, these genetic subgroups correlated well with clinical parameters, such as INSS stages, age, and overall survival (Figure 2B). The percentage of stage 4 patients in each group was much higher in groups A, B, C, and D (A: 70.2%, B: 71.2%, C: 84.1%, and D: 81.3%) compared to groups E and F (E: 24.4% and F: 32.6%) (*P* < 0.0001). On the other hand, the percentage of early-stage (stages 1 and 2) cases were significantly enriched in groups E and F (E: 33.3% and F: 43.5%) compared to other groups (A: 12.8%, B: 16.9%, and C: 3.4%). The age of patients was significantly higher in group D (median age, 43.0 months [range, 0–239]) than in the other groups (*P* < 0.0001), while it was significantly lower in group E (median age, 7 months [range, 0–264]) than in the other groups *(P* < 0.0001) (Table 1). The patients in groups A, B, C, and D had significantly poor prognoses, whereas those in groups E and F had favorable prognoses (*P* < 0.0001) (Figure 2B, Table 1). Group E was highly correlated with hyperdiploid lesions, which were observed in 87 of 123 cases (70.7%).

**Table 1:** Characteristics of each genetic subgroup

	Group A	Group B	Group C	Group D	Group E	Group F	*P* value
Number	47	59	88	91	123	92	
Median age	21.0	42.0	27.0	43.0	7.0	26.5	
[range](months)	[0–163]	[0–185]	[1–132]	[0–239]	[0–264]	[0–188]	<0.0001
Stage							
Stage 1–3, 4S	14	17	14	17	93	62	
Stage 4	33	42	74	74	30	30	<0.0001
Outcome							
Alive	25	38	45	64	104	78	
Dead	22	21	43	27	19	14	<0.0001

The systematic search for genomic alterations in large samples enabled us to detect significant correlations among different genetic lesions. Thus, we conducted pairwise comparisons across major driver events, such as *MYCN* amplification, *ALK* abnormalities, 1p LOH, and 11q LOH in our entire cohort (Figure 2C). *MYCN* amplification and 1p LOH showed the most significant positive correlation (FDR *q* < 10^−4^), followed by *MYCN* amplification and *ALK* mutation/amplification (FDR *q* < 10^–4^) and 1p LOH and *ALK* mutation/amplification (FDR *q* < 10^−3^) (Figure 2C). In contrast, 11q LOH, characterized by poor survival, significantly coexisted with 3p, 4p, 6q, and 19q LOH (FDR *q* < 10^−4^), suggesting that not only 11q LOH but also co-operation among these chromosomal losses contributed to the aggressive phenotype. On the other hand, as previously reported, *MYCN* amplification and 11q LOH showed a significant negative correlation (FDR *q* < 10^−3^), confirming the mutually exclusive phenotype of tumors characterized by *MYCN* amplification and 11q LOH (Figure 2C).

### Genetic and clinicopathological features of group A

**Figure 3:**
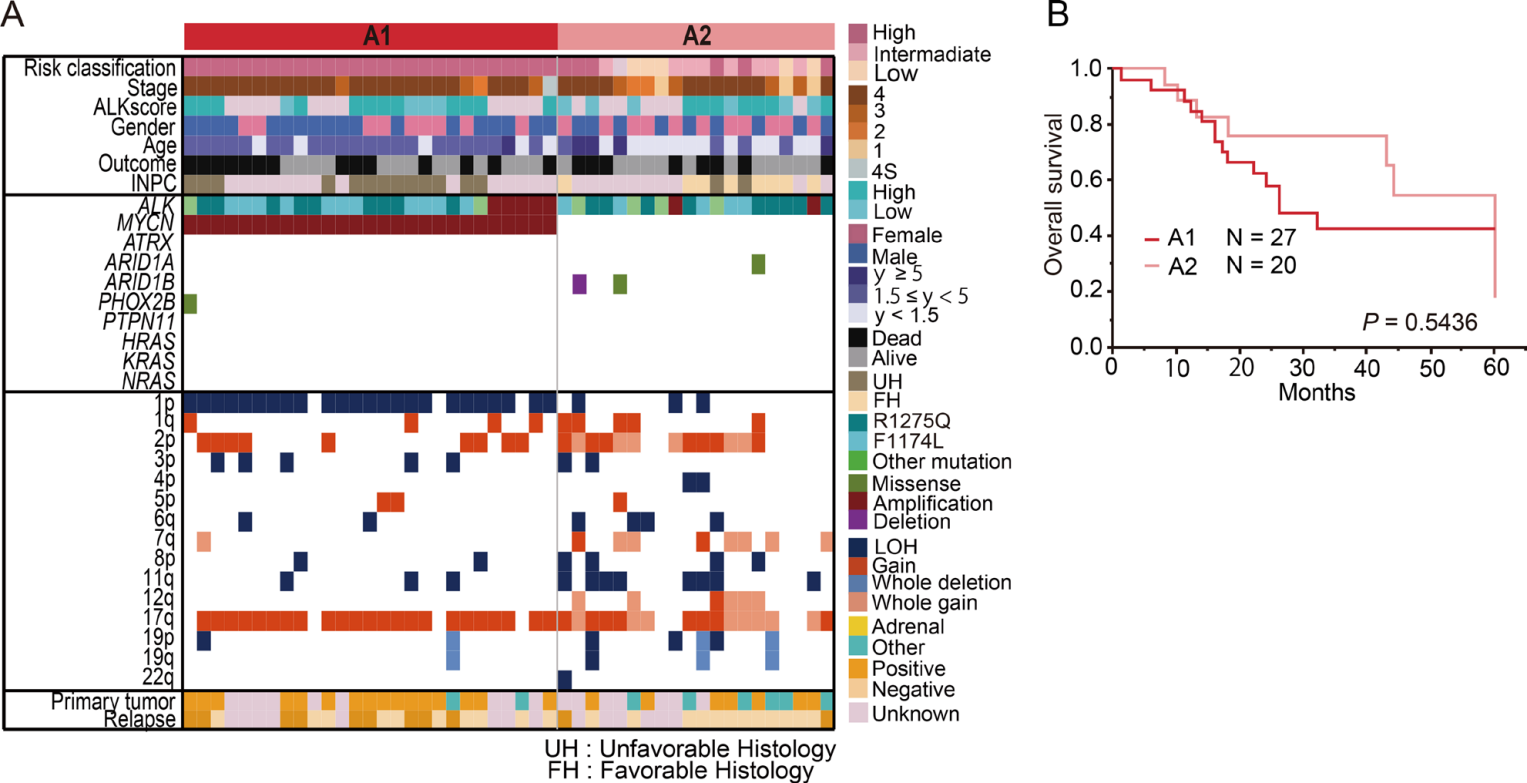
Genetic and clinical characteristics of group A (ALK mutation/amplification) (**A**) Landscape of genetic lesions in group A including 47 neuroblastomas with *ALK* mutation/amplification. According to the *MYCN* status, group A can be divided into the following 2 subgroups: group A1 (*ALK* abnormalities with *MYCN* amplification) and group A2 (*ALK* abnormalities without *MYCN* amplification). (**B**) Overall survival of neuroblastoma patients in groups A1 and A2.

To further analyze the genetic features of *ALK* abnormality-positive tumors, we classified group A into the following 2 subgroups based on the *MYCN* status: group A1 (*ALK* mutation/amplification with *MYCN* amplification; 57.4% of cases) and group A2 (*ALK* mutation/amplification without *MYCN* amplification; 42.6% of cases) (Figure 3A). Although gene mutations were less frequent in both groups A1 and A2, copy number alterations in various chromosomes, such as 1p LOH and 2p gain, 7q or whole chromosome 7 gain, 11q LOH, and 12q or whole chromosome 12 gain were significantly frequently detected (Supplementary Table 2). There was high concordance between *MYCN* amplification and 1p LOH in group A1, whereas a variety of copy number changes, including 7q or whole chromosome 7 gain and whole chromosome 17 gain were significantly gathered in group A2 compared to A1 (7q or whole 7 gain: *Pc* < 0.0001 and whole chromosome 17 gain: *Pc* = 0.0016) (Supplementary Table 2), suggesting that co-operating events in subgroup A tumors exhibited a highly genetic heterogeneity.

We further compared the differences in clinicopathological features between groups A1 and A2. Group A1 was significantly characterized by unfavorable histology (*P* < 0.0001), but the ALK score according to IHC analysis was not significantly different between these groups (*P* = 0.10). The prevalence of stage 4 cases was significantly higher in group A1 than in group A2, and patients in A1 tended to be older at diagnosis than those in A2. Additionally, primary adrenal site was more frequent in group A1 than in group A2 (*P* = 0.027). The relapse rate was slightly higher in group A1 than in group A2 (*Pc* = 0.96 by Chi-square test and *P* = 0.0615 by Fisher's exact test) (Supplementary Table 2); however, the survival rate was not statistically different between groups A1 and A2 (*P* = 0.54) (Figure 3B).

### Genetic characteristics of patients older than 5 years

**Figure 4:**
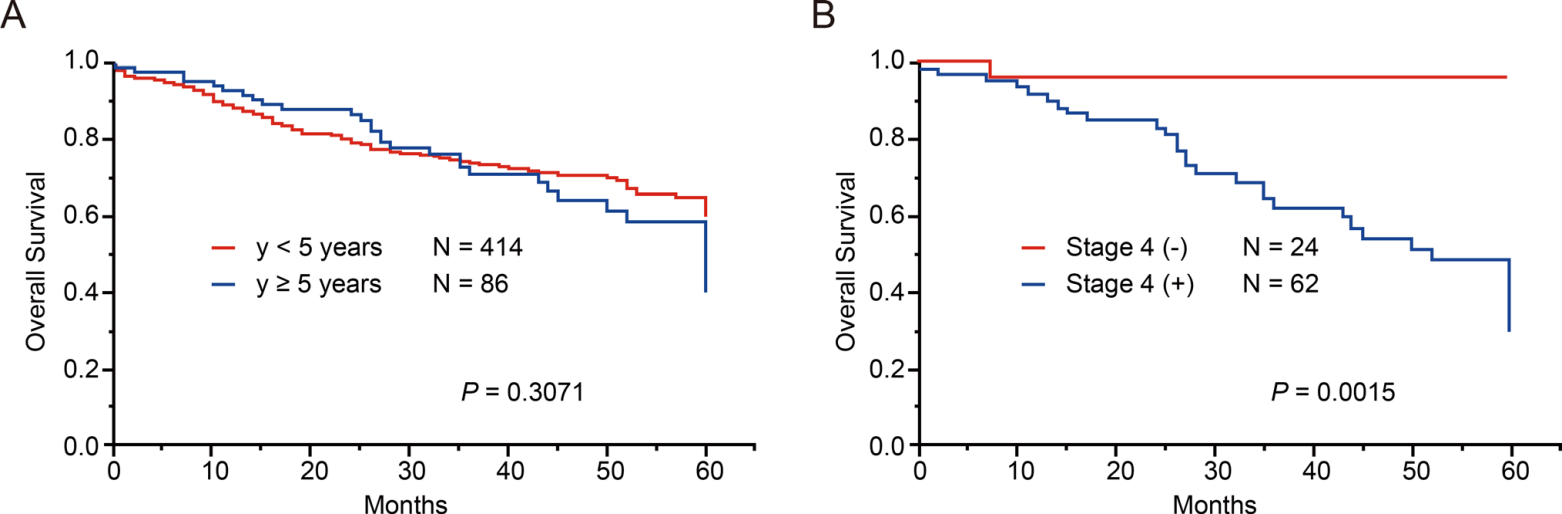
Overall survival of 500 neuroblastoma patients according to age at diagnosis and stages Overall survival according to age at diagnosis in neuroblastoma patients (**A**) and overall survival according to stage4 or non-stage4 in neuroblastoma patients older than 5 years (**B**).

**Figure 5:**
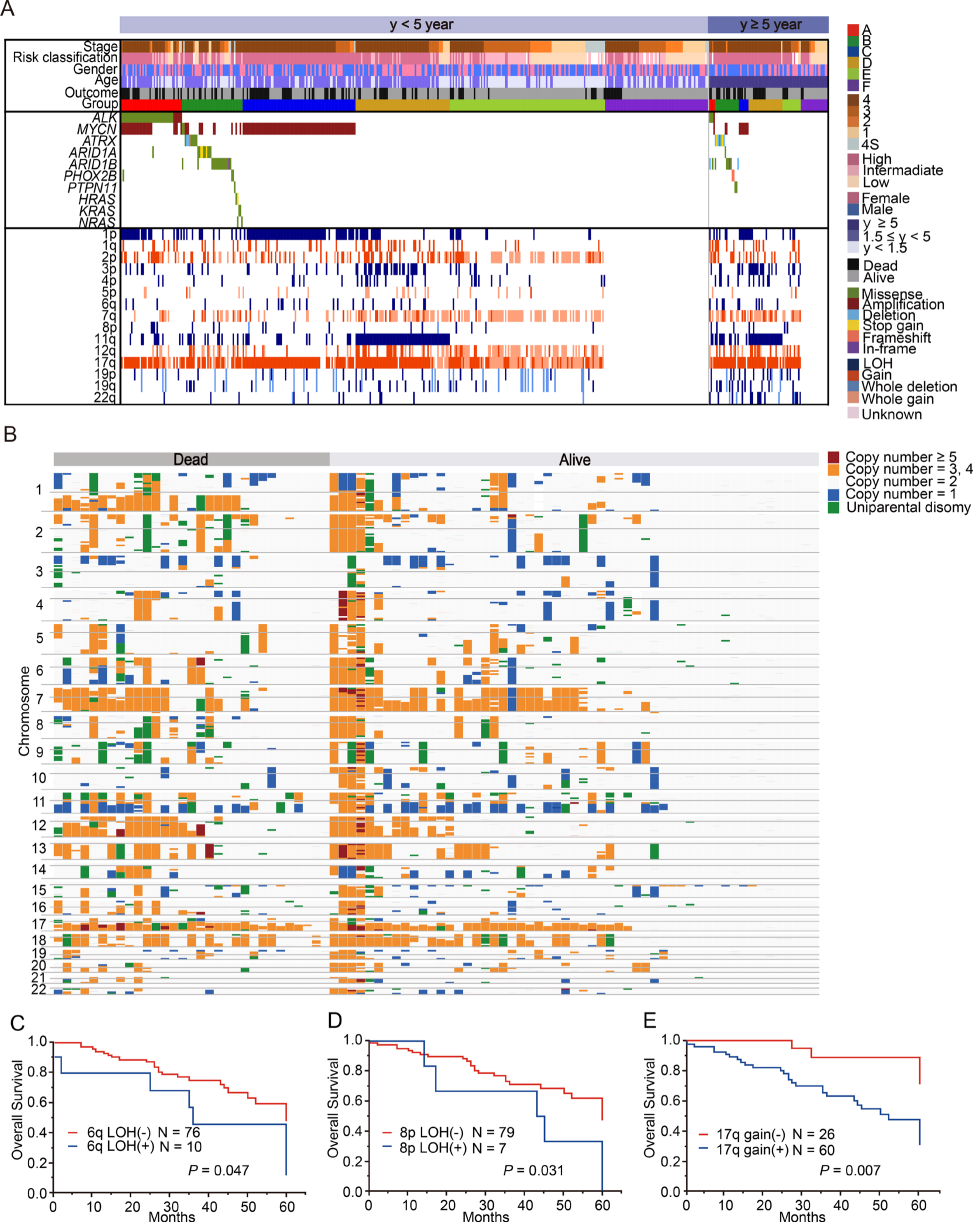
Characteristics of neuroblastoma patients older than 5 years and those younger than 5 years at diagnosis (**A**) Mutational and copy number change profiles of patients according to age at diagnosis. Among the patients aged under 5 years, group A included 10.1% of patients; group B, 13.0%; group C, 19.3%; group D, 16.0%; group E, 23.4%; and group F, 18.1%. On the other hand, among the patients aged over 5 years, group A included 5.8% of patients; group B, 19.8%; group C, 8.1%; group D, 29.1%; group E, 15.1%; and group F, 22.1%. (**B**) Overview of copy number changes and allelic imbalances detected using a single nucleotide polymorphism (SNP) array in 86 neuroblastoma patients older than 5 years at diagnosis. Overall survival curves according to 6q LOH (**C**), 8p LOH (**D**), and 17q gain (**E**) in the patients older than 5 years at diagnosis.

As 90% of neuroblastoma cases are diagnosed by age 5 years [23], cases over 5 years are very rare, and thus, the genetic characteristics of this group have been poorly studied. Therefore, we next investigated the genetic characteristics of 86 cases older than 5 years (older group) in our cohort (Supplementary Table 3). The older group was characterized by advanced stage of disease and high-risk classification; however, in our cohort, the overall survival was not significantly different between the younger (<5 years) and older groups (*P* = 0.31, Figure 4A). Similarly to younger group, non-stage 4 cases in order group showed excellent prognosis as represented in Figure 4B. Among the 86 cases in the older group, 67 (77.9%) had at least 1 genetic alteration, whereas 19 (22.1%) showed no genetic changes (Figure 5A). The most recurrent alterations in the older group were found in *MYCN* (amplification: 11.6%) followed by *ATRX* mutations (8.1%). In comparison to the younger group, *ATRX* mutations were obviously enriched in the older group (older: 8.1% vs. younger: 2.2%), whereas *MYCN* amplification was more frequent in the younger group (older: 11.6% vs. younger: 28.3%). The spectrum of CNVs in the older group was distinct from that in the younger group, except for 17q gain (72.1%) and 1p LOH (24.4%) (Figure 5B). The CNV signature of the older group was characterized by high frequencies of 3p LOH (26.7%), 4p LOH (15.1%), 11q LOH (43.0%), 19p LOH (15.1%), 19q LOH (15.1%), 22q LOH (22.1%), and 12q gain (18.6%) (Figure 5A, 5B, Supplementary Table 4). We further compared CNV signatures between alive and dead cases, but significant differences were not observed (Figure 5B). Of note, group E in the older group had distinct genetic features from group E in the younger group, with a low frequency of hyperploid cases (46.2%), and it exhibited unbalanced 17q gain combined with various partial CNVs (Figure 5A). Interestingly, *MYCN* amplification and 11q LOH were not significant risk factors for poor prognosis in the older group (*P* = 0.29 and *P* = 0.63), but 6q LOH, 8p LOH, and 17q gain were highly correlated with poor prognosis (*P* = 0.047, *P* = 0.031, and *P* = 0.007, respectively) in univariate analysis (Figure 5C–5E). However, in multivariate Cox regression analysis, only INSS stage 4 was found to independently affect overall survival (HR, 7.94; 95% confidence interval (CI), 1.63–143.23; *P* < 0.006) (Supplementary Table 5). Since LOH on 6q and 8p have been newly identified as genetic alterations related to poor prognosis in older patients, we further assessed the candidate genes in common regions of 6q and 8p LOH in older group. As a result, we found homozygous deletions involving *ARID1B* locus within minimal deleted region on 6q in 2 cases (Supplementary Figure 1). Furthermore, a total of 23 genes, including *CSMD1* gene (related to neuronal growth and cone stabilization), were detected in common region of 8p LOH (Supplementary Table 6). Since recurrent structural variants and a missense mutation of *CSMD1* gene have been found in neuroblastoma [14, 19], this gene may be the promising gene target for 8p LOH in neuroblastoma.

### Genetic characteristics of relapsed/refractory cases with neuroblastoma

**Figure 6:**
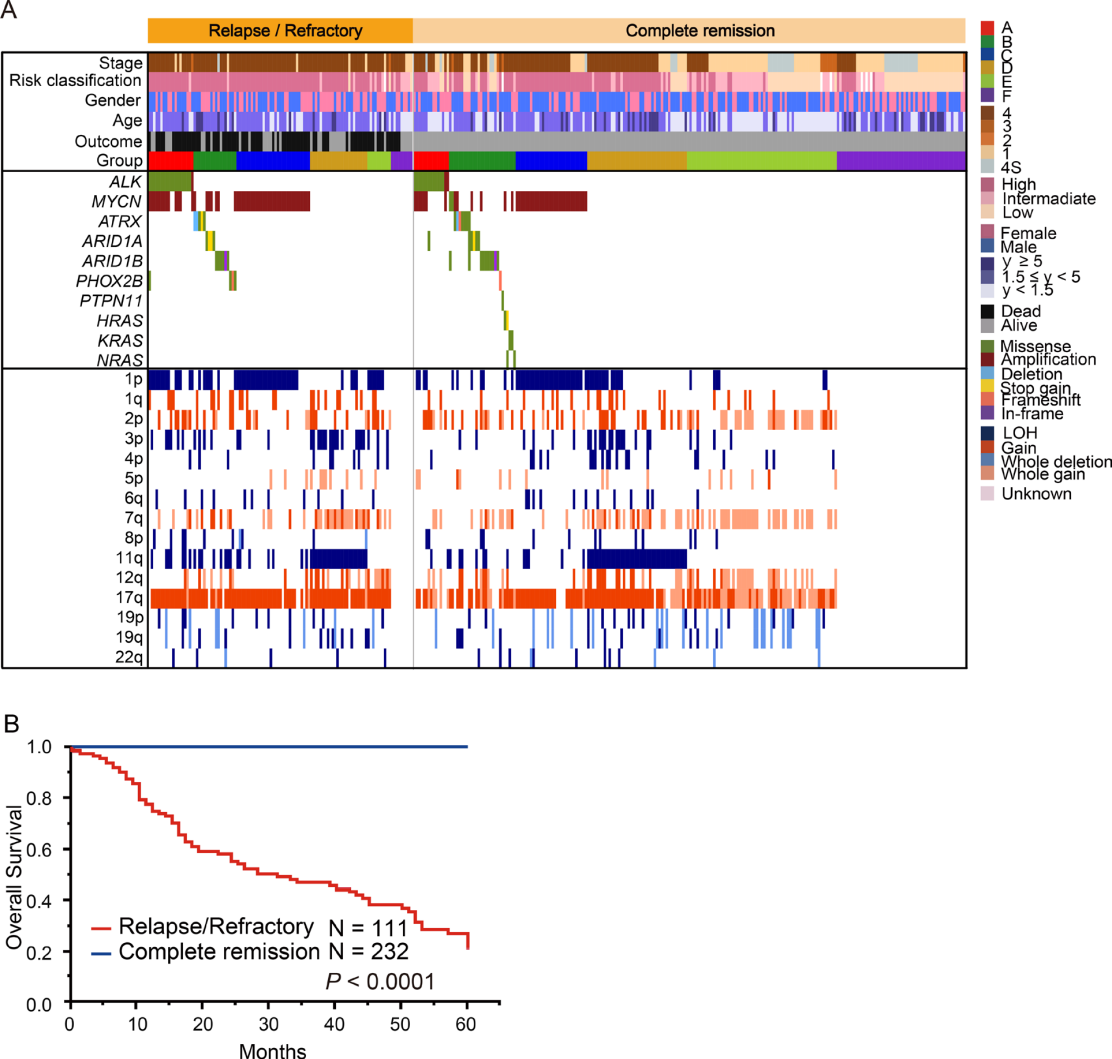
Comparison of the genetic and clinical landscape between 111 relapsed/refractory cases and 232 complete remission cases (**A**) Mutational and copy number change profiles of relapsed cases and non-relapsed cases among 343 neuroblastomas. Relapsed/refractory cases included 76 dead cases. This comparison excluded 157 patients without information about disease status. (**B**) Overall survival according to relapsed/refractory cases versus complete remission cases.

**Figure 7:**
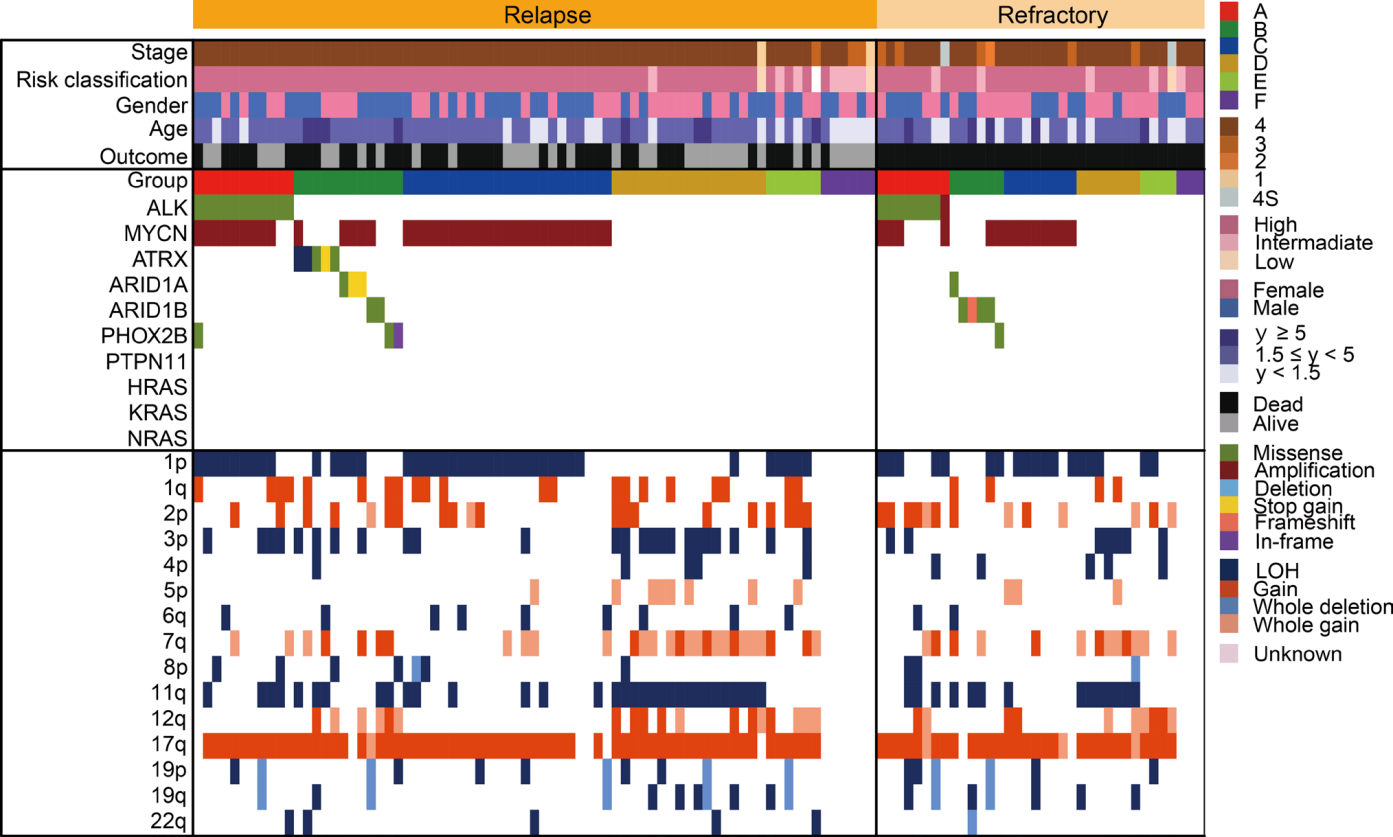
Comparison of the genetic and clinical landscape between 75 relapsed cases and 36 refractory cases Mutational and copy number change profiles of relapsed cases and refractory cases among 111 neuroblastomas.

**Figure 8:**
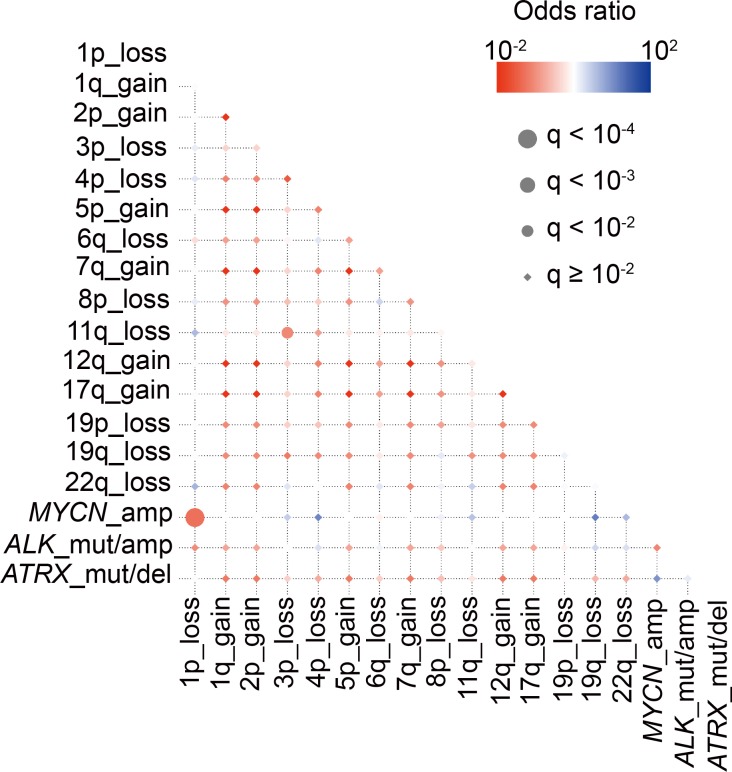
Correlations and temporal hierarchy of gene alterations in 75 neuroblastoma with relapse Statistically significant (*q* < 0.01) positive (red) and negative (blue) correlations among gene mutations/copy number changes.

Relapsed or primary resistant neuroblastoma patients often have a poor prognosis [1, 24], and the genetic features associated with relapsed/refractory disease have been incompletely described. Therefore, to reveal the genetic basis of relapsed/refractory neuroblastoma, we compared genetic characteristics between relapsed and/or refractory cases and non-relapsed cases. Among the 500 cases, 75 were relapsed, 36 were refractory, and 232 did not relapse. For the remaining 157 cases, we did not have disease status information. In our cohort, 76 of 111 (68.5%) cases in the relapsed/refractory group died, whereas all cases alive in the non-relapsed group (Figure 6A). Thus, relapsed/refractory cases had extremely poor prognosis compared to the non-relapsed cases (*P* < 0.0001) (Figure 6B). *ATRX* alterations and 1q gain were dominantly detected in the relapsed group, but the significant genetic differences between relapsed and refractory groups in our cohort were not observed (Figure 7 and Supplementary Table 7). As many as 50–60% cases with high-risk neuroblastoma patients will eventually suffer relapse [24], but our knowledge of relevant predictive genetic markers of relapse in neuroblastoma is still limited. Thus, in order to identify better predictive markers of relapse, we re-analyzed genetic changes between relapse and complete remission cases. In addition to the known risk factors, including stage, *MYCN* amplification, 1p and 3p LOH and 17q gain were significantly more frequently detected in relapsed cases than in complete remission cases (Supplementary Table 8). Moreover, significant coexistence of *MYCN* amplification and 1p LOH (FDR *q* < 10^−4^) and co-deletion of 3p and 11q (FDR *q* < 10^–2^) were observed in the relapsed group on pairwise comparisons (Figure 8). Of note, in multivariate analysis, the coexistence of *MYCN* amplification and 1p LOH (HR, 2.14; 95% CI, 1.03–4.51; *P* = 0.043), and the co-deletion of 3p and 11q LOH (HR, 2.60; 95% CI, 1.04–6.71; *P* = 0.040) were significant predictors of relapsed disease, and these were independent from clinical parameters (Supplementary Table 9). Although *MYCN* amplification, 17q gain, 1p LOH, and 3p LOH were correlated with relapse-free survival in univariate analysis, there was no significance in multivariate analysis, suggesting that combinations of these molecular parameters (*MYCN* amplification with 1p LOH and 3p/11q LOH) are important for the prediction of relapse (Supplementary Table 9).

### Relationship between ALK IHC staining and genetic abnormalities

**Figure 9:**
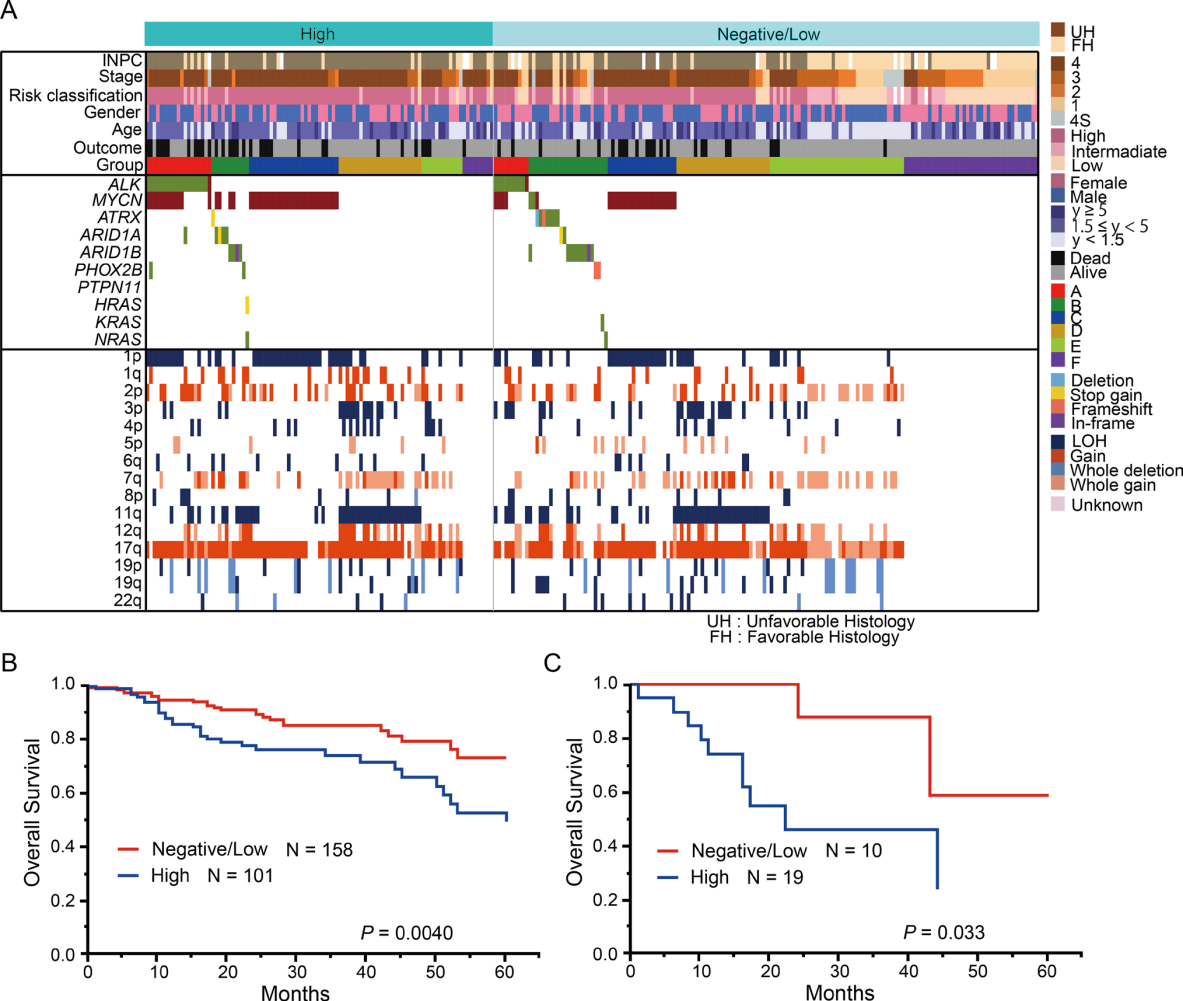
Genetic and clinical characteristics of the high and low ALK score groups (**A**) Genetic landscape of 259 neuroblastomas based on the ALK score. (**B**) Kaplan–Meier estimate for overall survival according to the ALK score. (**C**) Kaplan–Meier estimate for overall survival according to the ALK score in group A.

In addition to genetic alterations of *ALK*, elevated ALK expression levels have been previously reported in two-third of primary neuroblastoma cases [11, 25]. To assess the relationship between genetic lesions and ALK expression, IHC analysis of ALK was further performed in 259 cases. Among these 259 cases, 101 (39.0%) showed high ALK staining (ALK score +3 or +4; high ALK score), 101 (39.0%) showed low ALK staining (ALK score 1+ or +2; low ALK score), and 57 cases (22.0%) were negative (ALK score 0) (Figure 9A). Previous reports revealed that the ALK expression level is higher in neuroblastomas with *ALK* mutation/amplification than in tumors with wild-type *ALK* [26]. In accordance with this finding, the frequency of *ALK* mutation/amplification was significantly higher in the high ALK score group than in the negative/low ALK score group (*P* = 0.002). The 101 cases with a high ALK score were characterized by specific molecular (*MYCN* amplification; 1p, 3p, and 11q LOH; and 1q, 2p, and 17q gains), clinical (stage 4, relapsed disease, and primary adrenal site), and pathological parameters (INPC; unfavorable histology) (Supplementary Table 10). Accordingly, patients with high ALK score exhibited significantly poor prognosis compared to patients with negative/low ALK score (*P* = 0.004) (Figure 9B). The percentages of cases classified into genetic subgroups A and C were higher in the high ALK score group (18.8% and 25.7%, respectively) than in the negative/low ALK score group (6.3% and 13.9%, respectively) (*P* = 0.002 and *P* = 0.018, respectively). In contrast, the percentages of cases classified into groups E and F were higher in the negative/low ALK score group (23.4% and 26.0%, respectively) than in the high ALK score group (11.9% and 8.9%, respectively) (*P* = 0.018 and *P* = 0.0004, respectively). Furthermore, significant survival differences were observed between high ALK score and negative/low ALK score cases in group A (*P* = 0.033) (Figure 9C). However, no significant differences in the survival rate were detected in the other groups.

### The presence of 1p, 4p, and 11q LOH potentially contributed to the outcome prediction of intermediate-risk neuroblastoma

**Figure 10:**
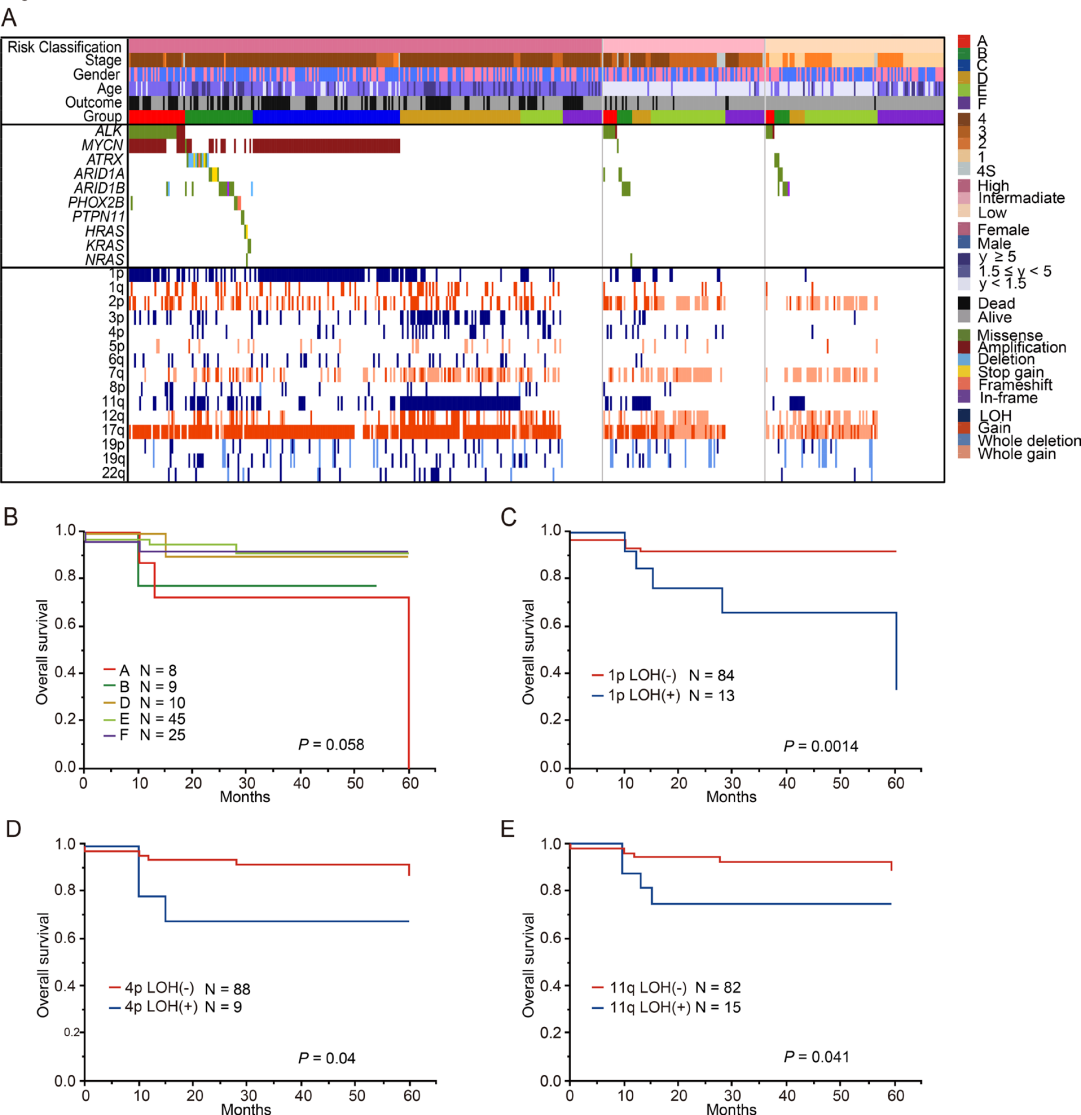
Genetic and clinical characteristics of the neuroblastoma patients classified intermediate risk group by COG risk classification (**A**) Mutational and copy number changes profiles by COG risk classification in 481 neuroblastomas. 19 patients without the information about COG risk classification excluded in this landscape. This cohort included 96 patients with intermediate-risk group. All patients with Group C (MYCN amplification) were in high-risk group. Overall survival for patients with 5 genetic subgroups in intermediate- risk group (**B**), 1p LOH in intermediate-risk group (**C**), 4p LOH in intermediate-risk group (**D**) and 11q LOH in intermediate-risk group (**E**).

During the last several decades, a body of literature regarding risk-based therapy for children with neuroblastoma has been accumulated [2]. Clinical stage, age, histopathology, *MYCN* copy number status, 11q LOH, and DNA ploidy are important for risk assessment in patients with neuroblastoma [27–31]. However, stratification is still imperfect, resulting in the under-treatment of few cases with intermediate risk. This may be due to the lack of systematic molecular characterization in this patient group of neuroblastoma. In our cohort, 97 cases were classified into the intermediate-risk group of COG risk classification, and we assessed the genetic features of these cases. In this intermediate-risk group, the proportion of patients in group E was the highest (46.4%), while the proportions of patients in groups A, B, and D were relatively low (8.2%, 9.3%, and 10.3%, respectively). To identify patients who might benefit the most from genetic alteration-based risk estimation, we analyzed the prognostic value of genetic changes in the intermediate-risk group (Figure 10A). There were no relationship between genetic subgroups and overall survival in patients with intermediated-risk group (Figure 10B). Intriguingly, in addition to 11q LOH (*P* = 0.041), 1p LOH and 4p LOH were significantly related to poor outcome (*P* = 0.0014 and *P* = 0.04, respectively) (Figure 10C–10E), although multivariate analysis did not show statistical significance (Supplementary Table 11).

## DISCUSSION

The present comprehensive genetic analysis revealed the whole features of the CNVs and mutation spectrum of major driver genes in a large series of neuroblastoma specimens. Based on genetic signatures, neuroblastoma patients can be divided into 6 molecular subgroups, which displayed different clinical characteristics, confirming the genetic diversity of neuroblastomas. Owing to the presence of very few genetic changes in tumors from group F, the majority of these tumors may be driven by epigenetic modifications, germline variants, or substantial numbers of mutations, which went undetected, as only 10 neuroblastoma-related genes were analyzed.

Our study revealed the following 2 subgroups of group A: one was a group having *ALK* abnormalities with *MYCN* amplification (group A1) and the other was a group without *MYCN* amplification (group A2). Each of these subgroups displayed different genetic backgrounds and clinical features, and there were no concordances with mutation types and these subgroups. Therefore, mutated *ALK* alleles may not be selected during the branching evolution of tumor development. Since the overall survival rate between groups A1 and A2 was not significantly different, *MYCN* amplification may not be a prognostic indicator in these groups. However, because the relapse rate was higher in group A1 than in group A2, the combined occurrence of *MYCN* amplification and *ALK* abnormalities is likely to contribute to the gain in metastatic progression capacities. In agreement with this, our previously reported *ALK^R1275Q^* knock-in and *MYCN* transgenic compound mice revealed that the co-operation of *ALK* mutation and *MYCN* overexpression results in impaired normal extracellular matrix/basement membrane integrity and enhanced tumor growth and dissemination [32]. Because *MYCN* amplification and *TERT* alterations were mutually exclusive [15–16], aggressive phenotype of group A2 might be correlated with high *TERT* expression. Thus, to provide an adequate assessment of molecular basis of group A2, further study is required.

Through IHC analysis and genetic screening, we demonstrated that overall survival was significantly worse in cases with a high ALK score than in those with a low ALK score in group A, indicating the reliable prognostic value of IHC analysis for ALK in cases with ALK abnormalities. Therefore, the combination of IHC findings and the genetic status of ALK can support therapeutic decisions.

In this cohort, patients who were older than 5 years at diagnosis had partial gains and deletions of chromosomes, typical 19p, 19q, and 22q LOH, and these alterations were more common in these patients than in those younger than 5 years. These findings indicated that the genetic background of neuroblastomas in children older than 5 years is distinct from that in children younger than 5 years. Because 19p/19q LOH and 22q LOH were commonly found in patients older than 5 years at diagnosis, analysis of these chromosomal aberrations might help elucidate the mechanisms of tumor development in patients older than 5 years. In addition, our results suggested that 6q and 8p LOH and 17q gain might be useful to predict the outcome of patients older than 5 years, although the number of patients with these chromosomal abnormalities was too small for detecting statistically independent associations between these CNVs and a patient's poor prognosis.

Previous papers have identified several driver oncogenic events, such as alterations in the *RAS/MAPK* pathway, in relapsed neuroblastomas, using high throughput sequencing [17]. However, as these gene alterations are enriched in only relapsed cases, the actionable genetic alterations at diagnosis that predict occurrence of relapse have been poorly studied, except for *MYCN* amplification [28]. In our study, we showed that the coexistence of *MYCN* amplification and 1p LOH, and the co-deletion of 3p and 11q in diagnostic samples were significantly correlated with the relapse rate. Therefore, it would be necessary to evaluate these genetic parameters at diagnosis in order to make the most appropriate therapeutic strategy.

Intermediate-risk neuroblastomas are thought to have high genetic diversity, and only limited data exist on prognostic molecular markers in this group. According to our genetic study, not only 11q LOH but also 1p and 4p LOH likely correlate with poor prognosis, and thus, these alterations may potentially contribute to relevant treatment stratification of the intermediate-risk group.

Our comprehensive genetic overview clarifies the clinical impact of genetic signatures and aids in the better understanding of copy number alteration spectrum of neuroblastoma. These findings provide important therapeutic opportunities, and they will help in the development of personalized medicine for neuroblastoma patients who still have a poor prognosis. However, since our genetic landscapes are drawn in a single cohort and lack information of *TRET* abnormalities, to provide accurate evaluations for molecular subgroups, further studies are needed.

## MATERIALS AND METHODS

### Patients and tumor samples

This study was approved by the ethics review board of the University of Tokyo (approved number: 1598). Neuroblastoma samples were obtained at the time of initial diagnosis from patients who had been diagnosed with neuroblastomas and admitted to Tokyo University Hospital and many other hospitals in Japan between 2003 and 2015. A total of 500 samples were assessed in this study. Among the 500 samples, 282 were obtained from the JNBSG. Informed consent was obtained from parents according to the relevant Japanese laws on the protection of persons taking part in biomedical research (Supplementary Table 3). All the tumors were classified according to the International Neuroblastoma Staging System (INSS) [33]. Risk stratification was performed according to Children's Oncology Group (COG) [1, 2]. Patients were treated according to several protocols [34, 35]. The *MYCN* gene copy number was determined as a routine diagnostic approach using FISH analysis combined with SNP array data in this study. The characteristics of the 500 study patients are summarized in Supplementary Table 3. DNA was extracted according to the manufacturer's instructions.

### Targeted deep sequencing of PCR-amplified fragments

Targeted deep sequencing for selected neuroblastoma-related genes (*ALK*, *MYCN, ATRX*, *ARID1A/1B*, *PTPN11*, *PHOX2B*, *HRAS*, *KRAS*, and *NRAS*) was performed using NotI-tagged primers (Supplementary Table 12). In addition to genes confirmed to be recurrently mutated in previous neuroblastoma sequencing studies, such as *ALK*, *ATRX*, *ARID1A*, *ARID1B*, *MYCN*, and *PTPN11*, we selected targeted genes directly involved in the *RAS* pathway (*NRAS*, *HRAS*, and *KRAS*). We also selected *PHOX2B*, which has been detected in patients with presumed genetic predisposition to neuroblastoma [13, 14, 19, 36–38]. The digested PCR product was purified, concatenated with T4 DNA ligase, and sonicated to generate fragments with an average size of 200 bp using a Covaris system. Within each genomic DNA pool, individuals with mutations were confirmed by subsequent sequencing by Hiseq 2000 or Miseq. Fragments were processed for sequencing according to a modified Illumina paired-end library protocol, and sequences were read with HiSeq 2000 or Miseq using a 100-bp or 150-bp paired-end read protocol [39]. Sequencing reads were aligned to hg19 using Burrows-Wheeler Aligner (BWA) with default parameters. The allele frequencies of SNVs and indels were calculated at each genomic position by enumerating the relevant reads with SAM tools. All variants showing VAF > 0.02 were extracted and annotated using ANNOVAR [40]. We excluded all variants found in dbSNP131, 1000 Genomes Project, and our in-house database, unless they were registered in the Catalogue of Somatic Mutations in Cancer (COSMIC version 60) [41]. The effects of the mutations on protein function were assessed using SIFT [42], PolyPhen-2 [43], and Mutation Taster [44]. To validate putative genomic variants detected in pooled sequencing, independent targeted deep sequencing was performed.

### SNP array analysis

We analyzed the genome-wide copy number variations (CNVs) for the 500 samples using GeneChip Human Mapping 250k Nspl or CytoScan HD arrays (Affymetrix) according to the manufacturer's instructions. CNAG/AsCNAR was used for subsequent informatics analysis for SNP array data as described previously [45, 46], which enabled accurate detection of allelic status without paired normal DNA, even in the presence of up to 70–80% normal cell contamination. Amplification was defined as a copy number of ≥5, gain was defined as a copy number of 3–4, and loss was defined as a copy number of 1. The array data have been deposited in the Japanese Genotype-phenotype Archive (JGA) at DNA Data Bank of Japan Center (DDBJ Center; http://www.ddbj.nig.ac.jp) with accession number JGAS00000000046 [47].

### IHC detection of ALK

IHC detection of ALK was performed using the anti-ALK mouse monoclonal antibody (Clone 5A4, Nichirei) and formalin-fixed paraffin-embedded tumor tissue [12]. Scoring (taking into account staining intensity and percentage positivity, modified from Passoni *et al* [25].) was performed as follows: 0, no staining (negative); 1+, weak cytoplasmic staining, <20% of cells (low); 2+, heterogeneous with low to moderate intensity of cytoplasmic staining, 20–50% of cells (low); 3+, heterogeneous with moderate to high intensity of cytoplasmic staining, 50–75% of cells (high); 4+, strong intensity of cytoplasmic staining, >75% of cells (high).

### Statistical analysis

Statistical analyses were performed using JMP^®^Pro11 (SAS Institute Inc., Cary, NC, USA). Correlations between clinical and molecular data were assessed by using the chi-square test. Relapse-free survival and overall survival, indicated with standard deviation, were estimated using the Kaplan–Meier method and were compared using the log-rank test. A *P*-value < 0.05 was considered to indicate significance. Relapse-free survival was calculated from diagnosis until the date of relapse. Overall survival was calculated from diagnosis until last follow-up or disease-related death. For multivariate analysis, we applied Cox proportional hazards regression models based on event-free survival with a backward procedure. Correlations between mutations and CNVs were investigated using Chi-squared test. The *Pc* values were adjusted by using Bonferroni's correction for multiple comparisons. Mann-Whitney *U*-Test was used for comparison of age at diagnosis between groups A1 and A2. We performed exhaustive pairwise testing across 3 genes (*ALK MYCN*, and *ATRX*) and 16 copy number changes in chromosome arms. Multiple testing was corrected using the method proposed by Benjamini-Hochberg [48, 49], which was considered statistically significant at a *P*-value < 0.01 [50].

## SUPPLEMENTARY MATERIALS FIGURES AND TABLES





## Abbreviations

ALK: Anaplastic lymphoma kinase
MYCN: V-Myc Avian Myelocytomatosis Viral Oncogene Neuroblastoma Derived Homolog
TERT: Telomerase Reverse Transcriptase
FDR: False discovery rate
COG: Children's Oncology Group
JNBSG: Japan Neuroblastoma Study Group
