# Supplementary material for: Identification of the genetic and clinical characteristics of neuroblastomas using genome-wide analysis
Source: Oncotarget. 2017 Nov 18;8(64):107513–29. doi: 10.18632/oncotarget.22495 (PMC5746085; doi:10.18632/oncotarget.22495)
Supplement: Supplementary file 1 [file oncotarget-08-107513-s001.pdf]

Identification of the genetic and clinical characteristics of neuroblastomas using genome-wide analysis

SUPPLEMENTARY MATERIALS

Supplementary Table 1: List of detected mutations by targeted amplicon sequencing

Gene	Location	Mutation Type	Refseq	Amino acid change	Position	Allele change	Frequency
ALK	2p	Missense	NM_004304	p.R1275Q	29432664	C>T	17
ALK	2p	Missense	NM_004304	p.F1174L	29443695	G>T	14
ALK	2p	Missense	NM_004304	p.F1174V	29443697	A>C	2
ALK	2p	Missense	NM_004304	p.T1151M	29443631	G>A	1
ALK	2p	Missense	NM_004304	p.T1087L	29446307	G>A	1
ALK	2p	Missense	NM_004304	p.L1196M	29443631	G>T	1
ALK	2p	Missense	NM_004304	p.F1245L	29436858	G>T	1
ALK	2p	Missense	NM_004304	p.R1192P	29443642	C>G	1
ALK	2p	Missense	NM_004304	p.F1245C	29436859	A>C	1
MYCN	2p	Missense	NM_001293228	p.P44L	16082317	C>T	1
MYCN	2p	Missense	NM_001293228	p.E69G	16082392	A>G	1
ATRX	Xq	Missense	NM_138270	p.I536V	76939028	T>C	2
ATRX	Xq	Nonsense	NM_138270	p.E1871X	76855262	C>A	1
ATRX	Xq	Missense	NM_138270	p.S2148Y	76813064	G>T	1
ATRX	Xq	Frameshift	NM_138270	p.P1333fs	76918878	TC>-	1
ATRX	Xq	Missense	NM_138270	p.Q2145H	76813072	C>A	1
ATRX	Xq	Nonsense	NM_138270	p.R2073X	76814313	G>A	1
ATRX	Xq	Missense	NM_138270	p.D1013H	76937597	C>G	1
ATRX	Xq	Missense	NM_138270	p.T2162P	76813023	T>G	1
ATRX	Xq	Missense	NM_138270	p.I1170T	76937125	A>G	1
ARID1A	1p	Missense	NM_006015	p.A221V	27023556	C>T	1
ARID1A	1p	Missense	NM_006015	p.D2129E	27106776	C>A	1
ARID1A	1p	Missense	NM_006015	p.P1521S	27101279	C>T	1
ARID1A	1p	Missense	NM_006015	p.I1691V	27102145	A>G	1
ARID1A	1p	Missense	NM_006015	p.S303T	27023802	G>C	1
ARID1A	1p	Missense	NM_006015	p.N874S	27089665	A>G	1
ARID1A	1p	Nonsense	NM_006015	p.Q200X	27023492	C>T	1
ARID1A	1p	Nonsense	NM_006015	p.E2246X	27107125	G>T	1
ARID1A	1p	Missense	NM_006015	p.G1761C	27105670	G>T	1
ARID1A	1p	Missense	NM_006015	p.D1196N	27099349	G>A	1
ARID1A	1p	Missense	NM_006015	p.G1016S	27094338	G>A	1
ARID1A	1p	Missense	NM_006015	p.Y471C	27057704	A>G	1
ARID1A	1p	Missense	NM_006015	p.N874S	27089665	A>G	1
ARID1A	1p	Missense	NM_006015	p.P1771S	27105700	C>T	1
ARID1A	1p	Nonsense	NM_006015	p.S1138X	27098997	C>A	1
ARID1B	6q	Missense	NM_017519	p.Q912H	157469981	G>T	1
ARID1B	6q	Missense	NM_017519	p.T1819S	157527770	C>G	1
ARID1B	6q	Missense	NM_017519	p.A471T	157100474	G>A	1
ARID1B	6q	Missense	NM_017519	p.Q83H	157099312	G>T	1
ARID1B	6q	Missense	NM_017519	p.Q1709H	157527441	G>T	2
ARID1B	6q	Nonframeshift	NM_017519	p.A445	157100396	*>+CGC	1
ARID1B	6q	Missense	NM_017519	p.R1789Q	157527680	G>A	1
ARID1B	6q	Missense	NM_017519	p.M2119V	157528669	A>G	1
ARID1B	6q	Missense	NM_017519	p.L64P	157099254	T>C	1
ARID1B	6q	Missense	NM_017519	p.M960T	157488212	T>C	1
ARID1B	6q	Missense	NM_017519	p.S49F	157099209	C>T	4
ARID1B	6q	Missense	NM_017519	p.P488L	157100526	C>T	1
ARID1B	6q	Missense	NM_017519	p.N1659S	157525120	A>G	1
ARID1B	6q	Missense	NM_017519	p.A1993V	157528292	C>T	1
ARID1B	6q	Nonframeshift	NM_017519	p.Q120	157099423	*>+CAA	1
ARID1B	6q	Missense	NM_017519	p.A460V	157100442	C>T	1
ARID1B	6q	Missense	NM_017519	p.K1612N	157522603	G>T	1
ARID1B	6q	Missense	NM_017519	p.Q563L	157150506	A>T	1
PHOX2B	4p	Missense	NM_003924	p.S311N	41747837	C>T	1
PHOX2B	4p	Missense	NM_003924	p.K168R	41748266	T>C	1
PHOX2B	4p	Missense	NM_003924	p.D177E	41748238	G>C	1
PHOX2B	4p	Frameshift	NM_003924	p.G268fs	41747966	C>-	2
PTPN11	12q	Missense	NM_002834	p.G503A	112926888	G>C	2
PTPN11	12q	Nonframeshift	NM_002834	p.59-60del	112888159	ACTGGT>-	1
HRAS	11p	Missense	NM_001130442	p.G12S	534289	C>T	1
HRAS	11p	Nonsense	NM_001130442	p.Q70X	533848	G>A	1
KRAS	12p	Missense	NM_004985	p.K117N	25378647	T>G	1
KRAS	12p	Missense	NM_004985	p.G12V	25398284	C>A	1
NRAS	1p	Missense	NM_002524	p.C51R	115256560	A>G	1
NRAS	1p	Missense	NM_002524	p.A59D	115256535	G>T	1

Supplementary Table 2: Comparison of genetic and clinical characteristics between groups A1 and A2

	A1<br>No. (%)	A2<br>No. (%)	X^2 Test<br>**Pc* value
Total	27	20	
1p LOH	24 (88.9)	3 (15.0)	<0.0001
1q gain	4 (16.7)	5 (25.0)	1.00
Whole 2 gain	9 (33.3)	13 (65.0)	0.72
2p gain	9 (33.3)	7 (35.0)	1.00
3p LOH	4 (14.8)	2 (10.0)	1.00
4p LOH	0 (0)	2 (10.0)	1.00
5p gain	2 (7.4)	1 (5.0)	1.00
6q LOH	2 (7.4)	4 (20.0)	1.00
7q or whole 7 gain	1 (3.7)	8 (40.0)	0.029
8p LOH	2 (7.4)	4 (20.0)	1.00
11q LOH	3 (11.1)	8 (40.0)	0.48
12q or whole 12 gain	0 (0)	6 (30.0)	0.017
17q gain	23 (85.2)	8 (40.0)	0.026
Whole 17 gain	0 (0)	8 (40.0)	0.0016
19p LOH	1 (3.7)	3 (15.0)	1.00
19p or whole19 loss	2 (7.4)	5 (25.0)	1.00
19q LOH	0 (0)	1 (5.0)	1.00
19q or whole 19 loss	1 (3.7)	3 (15.0)	1.00
22q LOH	0 (0)	1 (5.0)	1.00
Stage 4	22 (81.5)	11 (55.0)	1.00
INPC	FH:0 UH:13	FH:8 UH:2	0.00022
Relapsed cases	9 (47.4)	2 (14.3)	0.96
Primary: adrenal glands	16 (88.9)	7 (53.8)	0.65
Age at diagnosis: median	29 month	14.5 month	1.00†

*The *Pc* values were adjusted by using Bonferroni's correction for multiple comparisons.
†The *P* values were calculated by Mann–Whitney *U*-Test. LOH: loss of heterozygosity.

Supplementary Table 3: Characteristics of 500 neuroblastoma patients

Characteristics		No. (%)
Total		500
Stage	1	65 (13.0)
	2	44 (8.8)
	3	86 (17.2)
	4	283 (56.6)
	4S	22 (4.4)
Gender	M	284 (56.8)
	F	216 (43.2)
Age	y < 1.5 years	187 (37.4)
	1.5 years ≤ y < 5 years	227 (45.4)
	y ≥ 5 years	86 (17.2)
Follow up period	Medians (range)	29 months (0–60)
Outcome	Alive	355 (71.0)
	Dead	145 (29.0)

Supplementary Table 4: Comparison of genetic and clinical characteristics between neuroblastoma patients with age y < 5 years and y ≥ 5 years

	y < 5 years No. (%)	y ≥ 5 years No. (%)	X^2 Test *Pc value
Total	414	86	
1p LOH	130 (31.4)	21 (24.4)	1.00
1q gain	50 (12.1)	16 (18.6)	1.00
2p gain	79 (19.1)	21 (24.4)	1.00
3p LOH	56 (13.5)	23 (26.7)	0.070
4p LOH	31 (7.5)	13 (15.1)	0.612
5p gain	32 (7.7)	6 (7.0)	1.00
6q LOH	25 (6.0)	10 (11.6)	1.00
7q or whole 7 gain	142 (34.3)	39 (45.5)	0.99
8p LOH	22 (5.3)	7 (8.1)	1.00
11q LOH	104 (25.1)	37 (43.0)	0.020
12q gain	42 (10.1)	16 (18.6)	0.63
12q or whole 12 gain	122 (29.5)	25 (29.1)	1.00
17q gain	305 (73.7)	62 (72.1)	1.00
19p LOH	20 (4.8)	13 (15.1)	0.031
19q LOH	24 (5.8)	13 (15.1)	0.11
22q LOH	12 (2.9)	19 (22.1)	<0.0001
Relapsed cases	66 (22.5)	8 (18.4)	1.00
Primary: adrenal glands	207 (71.9)	34 (66.7)	1.00

*The *Pc* values were adjusted by using Bonferroni's correction for multiple comparisons. LOH: loss of heterozygosity.

Supplementary Table 5: Multivariate analysis for the effects of genetic and clinical factors on overall survival of neuroblastoma patients with over 5 years

	No.	Hazard ratio	*P* value	95% CI
6q LOH	10	1.44	0.40	0.60–3.13
8p LOH	7	1.45	0.44	0.53–3.39
17q gain	60	2.75	0.064	0.95–11.69
Stage 4	62	7.94	0.0060	1.63–143.23

LOH: loss of heterozygosity.

Supplementary Table 6: Twenty-three genes in the common region of 8p LOH in older patients

Gene name	Ref Seq	Start	End
OR4F21	NM_001005504	116085	117024
RPL23AP53	NR_003572	158344	182318
ZNF596	NM_001042415	182459	197340
FAM87A	NR_103537	325930	333174
FBXO25	NM_012173	356807	419875
TDRP	NM_175075	439789	495781
ERICH1	NM_001303100	564736	681239
DLGAP2	NM_001346810	687718	1656642
LOC401442	NR_134292	688547	690374
LOC286083	NR_111948	1244293	1250823
DLGAP2-AS1	NR_103863	1513675	1569830
LOC101927752	NR_134303	1710128	1712750
CLN8	NM_018941	1703944	1734738
MIR3674	NR_037445	1749290	1749358
MIR596	NR_030326	1765396	1765473
ARHGEF10	NM_001308153	1772142	1906807
LOC101928058	NR_136274	1919344	1921511
KBTBD11-OT1	NR_126346	1919562	1924610
KBTBD11	NM_014867	1922044	1955102
MYOM2	NM_003970	1993155	2113475
MIR7160	NR_106983	2024668	2024720
LOC101927815	NR_125425	2387218	2585991
CSMD1	NM_033225	2792875	4852494

Supplementary Table 7: Comparison of genetic and clinical characteristics between relapsed and refractory neuroblastoma patients

	Relapse No. (%)	Refractory No. (%)	X^2 Test [*]*Pc* value
Total	75	36	
1p LOH	39 (52.0)	16 (44.4)	1.00
1q gain	21 (28.0)	4 (11.1)	0.771
2p gain	20 (26.7)	9 (25.0)	1.00
3p LOH	24 (32.0)	8 (22.2)	1.00
4p LOH	5 (6.7)	6 (16.7)	1.00
Whole 5 gain	11 (14.7)	3 (8.3)	1.00
6q LOH	9 (12.0)	2 (5.6)	1.00
7q or whole 7 gain	28 (37.3)	13 (36.1)	1.00
8p LOH	7 (9.3)	3 (8.3)	1.00
11q LOH	31 (41.3)	15 (41.7)	1.00
11q LOH with 3p LOH	15 (20.0)	4 (11.1)	1.00
12q gain	9 (12.0)	6 (16.7)	1.00
12q or whole 12 gain	18 (24.0)	10 (27.8)	1.00
17q gain	62 (82.7)	28 (77.8)	1.00
19p LOH	6 (8.0)	4 (11.1)	1.00
19q LOH	6 (8.0)	6 (16.7)	1.00
22q LOH	5 (5.4)	1 (2.8)	1.00
MYCN amp	37 (49.3)	13 (36.1)	1.00
MYCN amp with 1p LOH	31 (41.3)	11 (30.6)	1.00
Stage 4	70 (93.3)	29 (80.6)	1.00
Primary: adrenal glands	55 (79.7)	28 (82.4)	1.00

[*]The *Pc* values were adjusted by using Bonferroni's correction for multiple comparisons.
LOH: loss of heterozygosity and amp: amplification.

Supplementary Table 8: Comparison of genetic and clinical characteristics between neuroblastoma patients with relapse and complete remission

	Relapse No. (%)	CR No. (%)	X^2 Test [*]*Pc* value
Total	75	232	
1p LOH	39 (52.0)	58 (25.0)	<0.00042
1q gain	21 (28.0)	24 (10.3)	0.0084
2p gain	20 (26.7)	41 (17.7)	1.00
3p LOH	24 (32.0)	26 (11.2)	0.0013
4p LOH	5 (6.7)	21 (9.1)	1.00
Whole 5 gain	11 (14.7)	19 (8.2)	1.00
6q LOH	9 (12.0)	12 (5.2)	1.00
7q or whole 7 gain	28 (37.3)	75 (32.3)	1.00
8p LOH	7 (9.3)	14 (6.0)	1.00
11q LOH	31 (41.3)	56 (24.1)	0.11
11q LOH with 3p LOH	15 (20.0)	12 (5.2)	0.0063
12q gain	9 (12.0)	24 (10.3)	1.00
12q or whole 12 gain	18 (24.0)	60 (25.9)	1.00

17q gain	62 (82.7)	101 (43.5)	<0.0001
19p LOH	6 (8.0)	15 (6.5)	1.00
19q LOH	6 (8.0)	12 (5.2)	1.00
22q LOH	5 (5.4)	12 (5.2)	1.00
MYCN amp	37 (49.3)	43 (18.5)	<0.0001
MYCN amp with 1p LOH	31 (41.3)	38 (16.4)	0.00034
Stage 4	70 (93.3)	95 (41.0)	<0.0001
Primary: adrenal glands	55 (79.7)	154 (67.5)	0.987

[*]The *Pc* values were adjusted by using Bonferroni's correction for multiple comparisons. CR: complete remission, amp: amplification, and LOH: loss of heterozygosity.

Supplementary Table 9: Risk factors contributing to relapse of neuroblastoma identified by multiple logistic regression analysis

	No.	Risk ratio	*P* value	95% CI
1p LOH without *MYCN*	28	0.83	0.73	0.28–2.32
3p LOH without 11q LOH	12	1.82	0.42	0.40–7.79
11q LOH without 3p LOH	46	1.28	0.57	0.55–2.93
11q LOH with 3p LOH	27	2.60	0.040	1.04–6.71
11q LOH with 3p and 4p LOH	11	2.21	0.25	0.56–8.55
MYCN without 1p LOH	11	2.99	0.11	0.77–12.19
MYCN with 1p LOH	69	2.14	0.043	1.026–4.51
Stage 4	165	12.048	<0.0001	4.65–37.73

LOH: loss of heterozygosity.

Supplementary Table 10: Comparison of genetic and clinical characteristics between neuroblastoma patients with High ALK score and Low ALK score

	High (4,3) No. (%)	Low (2, 1, 0) No. (%)	X^2 Test [*]*Pc* value
Total	101	158	
1p LOH	48 (47.5)	38 (24.1)	0.0022
1q gain	19 (18.8)	14 (8.9)	0.46
2p gain	33 (32.7)	21 (13.3)	0.0044
3p LOH	17 (16.8)	26 (16.5)	1.00
4p LOH	14 (13.9)	9 (5.7)	0.572
5p gain	8 (7.9)	15 (9.5)	1.00
6q LOH	10 (9.9)	7 (4.4)	1.00
7q or whole 7 gain	36 (35.6)	46 (29.1)	1.00
8p LOH	7 (6.9)	11 (7.0)	1.00
11q LOH	40 (39.6)	44 (27.9)	1.00
12q gain	17 (16.8)	15 (9.5)	1.00
12q or whole 12 gain	29 (28.7)	33 (20.9)	1.00
17q gain	75 (74.3)	74 (46.8)	0.00022
19p LOH	9 (8.9)	10 (6.3)	1.00
19q LOH	6 (5.9)	12 (7.6)	1.00
22q LOH	5 (5.0)	8 (5.0)	1.00

Relapsed cases	29 (29.3)	27 (17.3)	0.572
Primary site: adrenal glands	75 (78.1)	95 (61.3)	0.11
INPC	FH:16 UH:81	FH:76 UH:71	<0.0001
MYCN amplification	42 (41.6)	25 (15.8)	0.00010
ALK mutation/amplification	19 (18.8)	10 (6.3)	0.048
Stage 4	81 (80.2)	71 (44.9)	<0.0001

[*]The *Pc* values were adjusted by using Bonferroni's correction for multiple comparisons.
LOH: loss of heterozygosity.

Supplementary Table 11: Multivariate analysis for overall survival in the patients with intermediate risk group

	No.	Risk ratio	*P* value	95% CI
1p LOH	13	3.87	0.051	1.00–14.26
4p LOH	9	2.49	0.25	0.49–10.14
11q LOH	15	1.017	0.98	0.20–4.43
Stage 4	34	4.29	0.044	1.041–21.21

LOH: loss of heterozygosity.

Supplementary Table 12: Primer sets for targeted amplicon sequencing. See Supplementary_Table_12

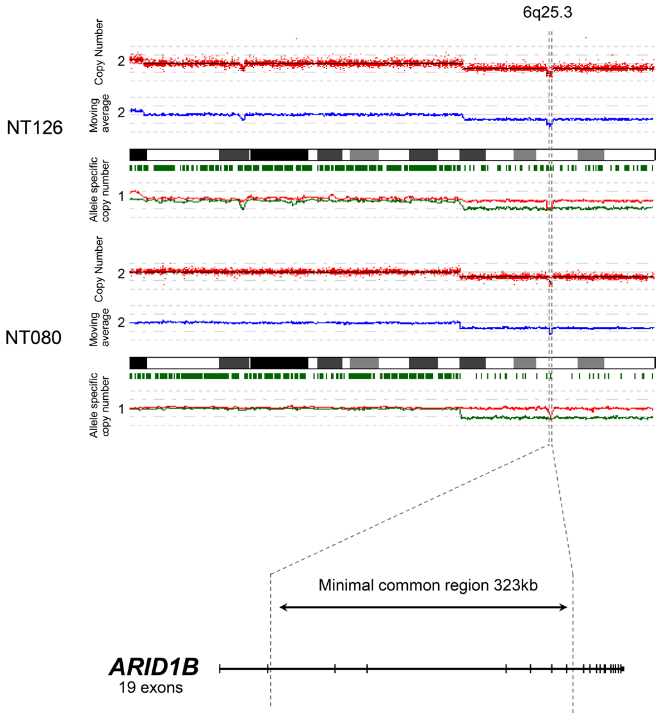

Supplementary Figure 1: ARID1B as a candidate tumor suppressor gene on chromosome 6q25.3 in neuroblastoma. Deletion mapping of 6q25.3 disclosed a homozygous deletion spanning a 320kb region in 2 samples (NT080 and NT126), which contains a part of ARID1B as the only structural gene. For each panel, total copy numbers (tCNs) (red dots), moving averages of tCNs for consecutive SNPs (blue line), an ideogram of the relevant chromosome, location of heterozygous SNP calls (green bars), and allele-specific copy numbers (AsCNs) averaged for consecutive SNPs (red and green lines for larger and smaller alleles, respectively) are plotted.
